# Exploring the “Multiple-Hit Hypothesis” of Neurodegenerative Disease: Bacterial Infection Comes Up to Bat

**DOI:** 10.3389/fcimb.2019.00138

**Published:** 2019-05-28

**Authors:** Kristin L. Patrick, Samantha L. Bell, Chi G. Weindel, Robert O. Watson

**Affiliations:** Department of Microbial Pathogenesis and Immunology, Texas A&M Health Science Center, Bryan, TX, United States

**Keywords:** pathogenesis, Parkinson's, Alzheimer's, neuroinflammation, Parkin (PARK2), LRRK2, PINK1, *Mycobacterium tuberculosis*

## Abstract

Despite major strides in personalized genomics, it remains poorly understood why neurodegenerative diseases occur in only a fraction of individuals with a genetic predisposition and conversely, why individuals with no genetic risk of a disorder develop one. Chronic diseases like Alzheimer's, Parkinson's, and Multiple sclerosis are speculated to result from a combination of genetic and environmental factors, a concept commonly referred to as the “multiple hit hypothesis.” A number of bacterial infections have been linked to increased risk of neurodegeneration, and in some cases, clearance of bacterial pathogens has been correlated with amelioration of central nervous system (CNS) deficits. Additionally, mutations in several genes known to contribute to CNS disorders like Parkinson's Disease have repeatedly been implicated in susceptibility to intracellular bacterial infection. Recent data has begun to demonstrate roles for these genes (*PARK2, PINK1*, and *LRRK2*) in modulating innate immune outcomes, suggesting that immune dysregulation may play an even more important role in neurodegeneration than previously appreciated. This review will broadly explore the connections between bacterial infection, immune dysregulation, and CNS disorders. Understanding this interplay and how bacterial pathogenesis contributes to the “multiple-hit hypothesis” of neurodegeneration will be crucial to develop therapeutics to effectively treat both neurodegeneration and infection.

## Introduction

Worldwide, an estimated 50 million people suffer from dementia/Alzheimer's Disease (AD), 7–10 million from Parkinson's Disease (PD), and over 2 million from Multiple sclerosis (MS). As our population ages, the incidence of neurodegenerative disease will continue to soar. The vast majority of neurodegenerative disease cases occur in people who have no known family history of the disorder. Current estimates state that only 15% of PD patients carry a mutation in a known Parkinson's-related factor (e.g., *LRRK2, PARK2*/Parkin, *PINK1*, or *SNCA*). While early-onset familial AD has a strong genetic component [e.g., mutations in amyloid precursor protein (*APP*), presenilin-1 (*PS1*), and presenilin-2 (*PS2*)], researchers have not identified genetic causes of late-onset AD, although the *APOE* e4 allele is associated with increased risk. Likewise, despite the identification of over 200 genes that may contribute to MS risk (e.g., *HLA-DRB1, IL7R*), the actual factors that precipitate demyelination of neurons and why these changes occur at a specific time in an individual's life remain unclear.

Clearly, factors beyond genetics are involved in precipitating and exacerbating diseases of the CNS. For decades, infections by viruses, bacteria, or eukaryotic parasites have been investigated as possible triggers for neurodegeneration. While many researchers remain skeptical of studies in human patients that suffer from small sample size and complicating co-morbidities, a preponderance of evidence strongly implicates an infectious component in neurodegenerative diseases. MS is perhaps the best described CNS disorder with a potential microbial trigger, as several latent viral infections, including herpes and Epstein-Barr, increase one's risk for MS. However, no infection has been identified as truly causal. A recent study that investigated “infection burden” in a cohort of PD patients and healthy controls measured antibody titers for a number of infections that have been associated with neurodegeneration including cytomegalovirus (CMV), Epstein Barr virus (EBV), herpes simplex virus (HSV-1), *Borrelia burgdorferi, Chlamydia pneumoniae*, and *Helicobacter pylori* (Bu et al., 2015). They reported that PD patients were statistically more likely to have been exposed to multiple pathogens relative to healthy controls. This likelihood was particularly robust when bacterial pathogens were assessed independently of viral infection. Along these same lines, other studies have found that vaccination against formerly common bacterial infections (diphtheria, tetanus, pertussis) associates with decreased risk for AD (Verreault et al., 2001).

**Figure 1:**
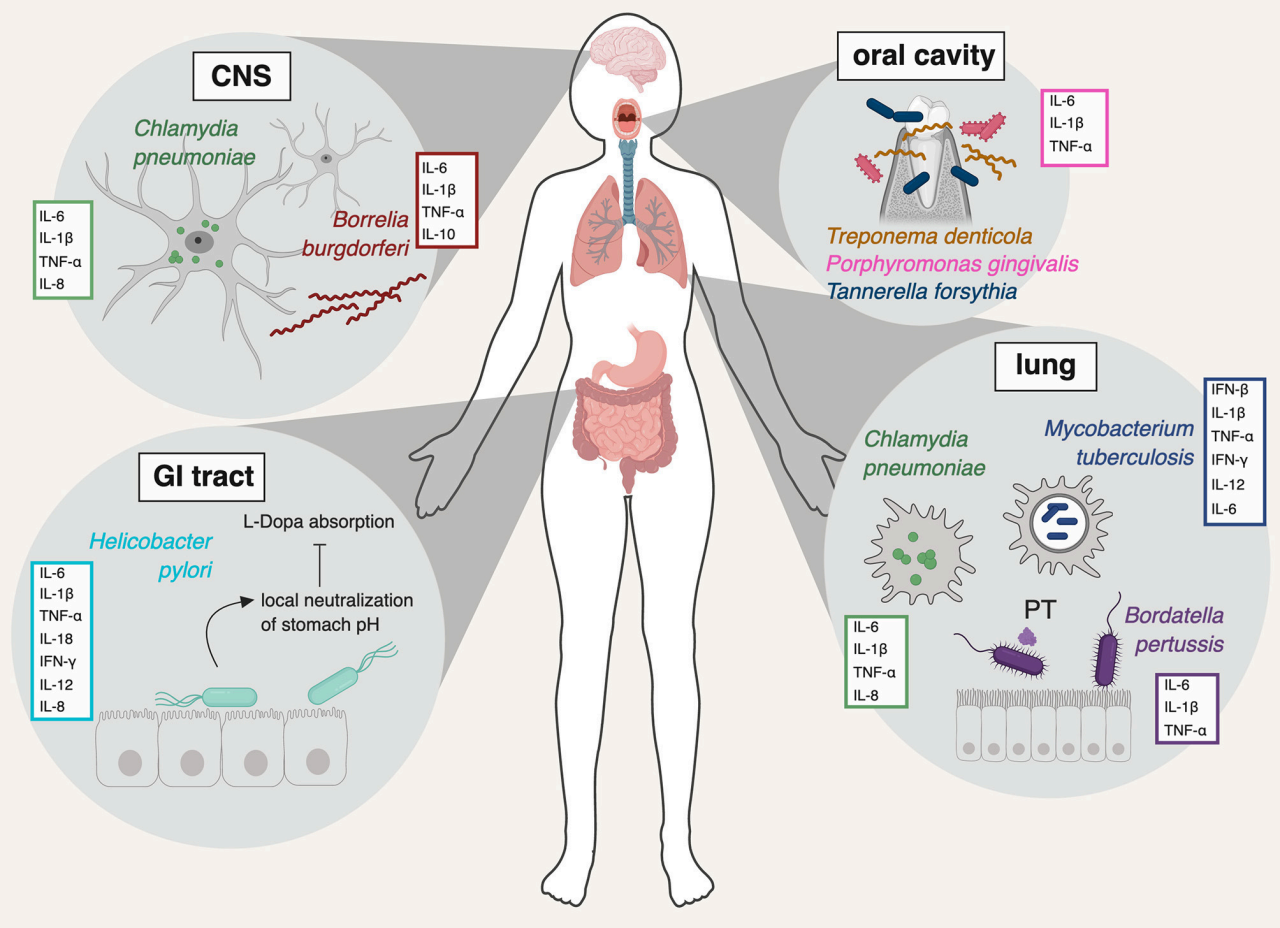
Schematic representation of the pathogens discussed in this review, the major organ system(s) they infect, and the primary innate immune cytokines they induce. *Chlamydia pneumoniae* is shown in its primary location (lung) but also in the CNS, where the bacterium has been repeatedly detected in association with AD (Balin et al., 1998; Arking et al., 1999; Gérard et al., 2006). *Borrelia burgdorferi* is a systemic infection, shown here in the CNS, where it too has been detected (Miklossy et al., 2004). *Helicobacter pylori* causes infection of the stomach and duodenum. *Mycobacterium tuberculosis* and *Bordatella pertussis* are mainly pathogens of the lung. The major resident oral pathogens, *Treponema denitcola, Porphyromonas gingivalis*, and *Tannerella forsythia* are shown in the mouth and gums. PT: pertussis toxin. Figure created with BioRender.

One obvious consequence of infection is peripheral inflammation and polarization of immune responses. While interest in neuroinflammation and how resident brain immune cells, like microglia and astrocytes, make and respond to cytokines and chemokines is growing, how these variables impact cells in the brain remains vastly understudied. Dysregulation of the gut microbiome has received a great deal of attention as a risk factor for CNS dysfunction and has been reviewed extensively (Zhu et al., 2017; Bell et al., 2018; Heiss and Olofsson, 2019). Here, we will focus on *pathogenic* bacterial species that have repeatedly been associated with three major neurodegenerative diseases: AD, PD, and MS (Figure 1). We will broadly describe the current evidence that supports a role for peripheral immune responses in eliciting AD, PD, and MS and highlight how specific bacterial pathogens have been linked to neurodegenerative diseases. Finally, as the links between PD and mycobacterial infections have been investigated with some mechanistic detail, we will explore the evidence that supports the “multiple-hit hypothesis” for PD.

### Local and Peripheral Immune Responses in Neurodegenerative Diseases

For decades, the CNS has been perceived as an immune privileged site, shut off from the peripheral immune response by virtue of the blood-brain barrier (BBB) and a lack of draining lymphatics. However, more recent data have repeatedly challenged this paradigm, showing that extensive mixing occurs between the CNS immune milieu and that of the periphery, with circulating proteins and metabolites able to access the brain. This shift in thinking allows us to seriously consider the idea that changes to the peripheral immune response can elicit neuroprotective and neurotoxic changes in the CNS.

#### Parkinson's Disease

PD is characterized by loss of dopaminergic neurons that originate in the substantia nigra (SNc) and deposit dopamine in the striatum. The striatum is a principle component of the basal ganglia, a group of neurons involved in coordinating voluntary movement. It serves as the reward and motor center of the brain. As these SNc-originating neurons deteriorate, dopamine levels in the striatum plummet, resulting in the motor deficits generally associated with PD (slow movement, tremors, rigidity). Because the striatum is also part of the brain's reward circuit, PD patients frequently suffer from depression and anxiety, likely due to decreased dopamine levels. The brains of PD patients accumulate protein deposits called Lewy bodies, which are mainly comprised of aggregates of the protein α-synuclein. Accumulation of α-synuclein deposits ultimately results in neuronal dysfunction. Levodopa, or L-3,4-dihydroxyphenylalanine, is a precursor to dopamine and the default treatment for PD-patients to improve parkinsonian symptoms in early disease stages. Levodopa is generally quite effective and well-tolerated but many patients experience response fluctuations as a consequence of irregular absorption and short drug half-life (Salat and Tolosa, 2013).

Approximately 95% of PD patients have idiopathic disease. The vast majority of these cases are likely caused by the combination of any number of extrinsic factors, including but not limited to infection, dysregulation of gut microbiota, and exposure to environmental toxins. Together, these stresses may directly damage healthy neurons, while also inducing expression of proinflammatory and cytotoxic factors by microglia and astrocytes. Over time, a positive feedback loop persists, whereby activation of these CNS resident immune cells damages neurons, thereby activating glial cells and inducing additional neuroinflammation.

Somewhat paradoxically, low levels of proinflammatory cytokines (IFN-β, IL-1β, IL-6, TNF-α) are absolutely required for the maintenance of a healthy brain and have been shown to aid in remodeling of neurocircuits and in neurogenesis (Yirmiya and Goshen, 2011). For example, *Ifnb*^−/−^ mice exhibit spontaneous behavioral and cognitive impairments and neurodegeneration. PD-like symptoms and dopaminergic neuron loss were preventable with IFN-β gene therapy (Ejlerskov et al., 2015). While this is indicative of a neuroprotective role for type I IFN, increased type I IFN expression has also been found in the brain tissue of post mortem PD patients, and type I IFN receptor (IFNAR) function is required in the 1-methyl-4-phenyl-1,2,3,6-tetrahydropyridine (MPTP) induced mouse model of PD (Main et al., 2016). These data imply that the dysregulation of type I IFN has diverse effects in PD progression, similar to its complex role in many other inflammatory conditions.

PD elicits strong innate and adaptive immune responses that can be detected in the CNS as well as in circulating serum. Two of the earliest biomarkers for PD include TNF-α and IL-1β. These cytokines, along with IL-2, IL-6, IL-8, CCL2, and IFN-γ, are elevated in the brains, cerebrospinal fluid (CSF), and blood of PD patients (Eidson et al., 2017; Schröder et al., 2018). A recent study showed that T cells from patients with PD recognize α-synuclein (Sulzer et al., 2017), and non-classical monocytes and reactive CD4+ and CD8+ reactive T cells are found in post-mortem brain tissue (Brochard et al., 2009; Sommer et al., 2018), indicating elevated inflammation as well as adaptive immune cell invasion into PD-patient brains. In the periphery, IFN-γ/IL-4 ratios are elevated in PD patients, demonstrating that the majority of reactive T cells are Th1 (Kustrimovic et al., 2018). Other reports have implicated pro-inflammatory Th17 cells in PD progression (Storelli et al., 2019). Together, these data strongly suggest an adaptive immune component to the pathophysiology of PD. PD may also have a humoral immune component, as increased IgG antibodies have been found against α-synuclein and dopaminergic neurons in patients with advanced disease. This suggests that antibodies are capable of crossing the BBB during active PD (Sanchez-Guajardo et al., 2013), but the contribution of IgG antibodies to earlier disease stages remains unclear (Scott et al., 2018). Because innate immune responses are critical in determining adaptive immune cell engagement and differentiation, it is possible that many of the T and B cell phenotypes described here occur downstream of innate immune responses following general stresses like mitochondrial damage or infection.

#### Alzheimer's Disease

AD is a disease of cognitive impairment rather than motor function deterioration. AD is characterized by synaptic loss in the cortex and hippocampus, alongside the presence of plaques and neurofibrillary tangles. These plaques are mainly comprised of amyloid beta peptide, which is derived by proteolytic cleavage of amyloid beta precursor protein (AβPP or APP). Gradual accumulation of these plaques correlates with synaptic loss and neuronal death. Likewise, neurofibrillary tangles are comprised of tau, a protein that associates with microtubules in mature neurons and helps stabilize the microtubule network. Hyperphosphorylated tau, which is seen in the brains of AD patients, is prone to oligomerize, which inhibits trafficking along microtubules and promotes formation of nonfunctional and toxic neurofibrillary tangles (Iqbal et al., 2010).

Like PD, infection and inflammation have been repeatedly associated with AD although the exact nature of these environmental factors remains unclear. Several lines of compelling evidence support a “multiple-hit hypothesis” for triggering AD. Notably, several AD risk-associated genes including *APP, APOE*, and *TREM2*, have been associated with immune activation, and a number of bacterial pathogens (e.g., *H. pylori, C. pneumoniae*, and *Porphyromonas gingivalis*) have been detected in the brains of AD patients (Price et al., 2006; Harris and Harris, 2015; Miklossy, 2015; Pisa et al., 2015; Carter, 2017). In addition, proinflammatory cytokines and immune cells have been implicated in AD disease progression. IL-1β has been shown to regulate the processing of APP, altering its levels (Li et al., 2003; Griffin et al., 2006; Tachida et al., 2008; Anderson et al., 2009). Similarly, IL-6, IL-18, IL-10, TNF-α, and TGF-β1 seem to play a role in APP metabolism and tau phosphorylation (Domingues et al., 2017). IFN-γ has been shown to promote phagocytosis of APP, thus promoting plaque clearance, but IFN-γ also induces production of IL-18 and IL-1β, which are associated with increased disease severity (Kim et al., 2000; Joosten et al., 2003). Treatment with TGF-β1 (Chen et al., 2015) or IFN-β (Mudò et al., 2019) are protective in rat models of AD and are thought to work by decreasing proinflammatory IL-1β, TNF-α, and iNOS, and by promoting anti-inflammatory IL-10. Both AD patients and AD animal models present high CD4+/CD8+ T cell ratios indicative of Th1 skewing in the periphery (Browne et al., 2013), and elevated levels of IL-6, IL-21, IL-22, IL-23 in the serum of AD patients point to a Th17 polarization in the periphery during early disease (Oberstein et al., 2018). Together, these data strongly implicate an immune component in triggering, exacerbating, and protecting against AD, and demand further investigation into how infection influences each of these phenomena.

#### Multiple Sclerosis

MS differs from PD and AD in that is an autoimmune disease whereby T cells attack the myelin sheath of neurons, preferentially in the optic nerve, brainstem, spinal cord, and subcortical areas of the brain. Activation of CNS-sensitized T cells damages not only myelin but also myelin-producing cells called oligodendrocytes and nerve fibers. Deterioration of myelin sets off an inflammatory process that triggers the recruitment of immune cells into the CNS and the release of inflammatory cytokines. Symptoms are varied but include muscle weakness, ataxia, visual problems, and acute or chronic pain. Symptomatic episodes can occur without warning, but common infections like influenza and gastroenteritis increase risk of relapse (Compston and Coles, 2008).

Interestingly, patients with MS have elevated circulating cytokines involved in chemotaxis including, CCL2, CCL3, CCL4, CXCL10, MIF, and Trail as well as IL-17 and IL-12(p40); many of these immune modulators have also been detected in patients' CSF (Khaibullin et al., 2017). These chemokines likely promote the infiltration of reactive T cells (IL-17-producing Th17 cells and GM-CSF-producing CD4+ and CD8+ T cells) into the brain, contributing to the death of neurons (Cao et al., 2015; Pierson and Goverman, 2017). Monocyte infiltration into the CNS also occurs, facilitated by IL-1β (Paré et al., 2018). The exact mechanism by which T cell populations or monocytes initially gain access to the brain is not well understood, but evidence points to roles IL-17 and IL-22 via breakdown of the BBB and modulation of resident immune cells of the brain (Kebir et al., 2007).

Type I and Type II IFNs seem to play paradoxical roles in MS, either promoting protection or pathogenesis depending on the stage of the disease (Arellano et al., 2015). In early stages of rodent MS, IFN-γ (type II IFN) produced by natural killer (NK) cells promotes a pro-inflammatory state by increasing M1 macrophages and promoting T cell infiltration into the brain, thus exacerbating disease (Dungan et al., 2014). In later stages of disease, however, IFN-γ actually attenuates inflammation (Furlan et al., 2001). Likewise, IFN-β (type I IFN) has been shown to decrease GM-CSF by CD4+ and CD8+ T cells, thereby limiting T cell infiltration into the brain, but it can also exacerbate disease in the absence of IFN-γ (Rasouli et al., 2015).

## Bacterial Pathogens Associated With Neurodegeneration

### Helicobacter pylori

*H. pylori* is a ubiquitous gram-negative, motile bacterium found in the stomach/upper gastrointestinal tract of approximately 50% of humans. Although the majority of infected individuals do not experience symptoms, *H. pylori* can cause gastritis and peptic ulcers and is associated with stomach cancer. Several adaptations are required for *H. pylori* to survive in and colonize the GI tract including secretion of urease, flagellar motility, and adhesion to the gastric epithelium.

*H. pylori* infection induces a pro-inflammatory innate immune response. One important way *H. pylori* induces inflammation is via the activity of genes in its *cag* pathogenicity island, which has been shown to mediate epithelial cell contact and promote IL-8 expression. Because its lipopolysaccharide (LPS) is a relatively weak inducer of toll-like receptor 4 (TLR4), most NFκB-dependent cytokine expression (e.g., IL-6, TNFα) is activated via TLR2 from its outermemberane lipoproteins. By virtue of it being a mucosal pathogen, mast cells and eosinophils are important cellular players in *H. pylori* gastritis (Ieni et al., 2016). Th1 cells are also critical as they dominate the T-cell response in the gastric mucosa and result in increased severity of gastritis (Robinson et al., 2007). This Th1-mediated response results in high levels of IFN-γ, TNF-α, and IL-12. *H. pylori* infection also promotes expression of IL-1 family cytokines, IL-1β and IL-18, due to inflammasome activation. Importantly, many of *H. pylori*-induced cytokines have been independently linked to exacerbation of PD (TNF- α, IFN- γ, IL-1β) and AD (IL-1β and IL-18) (Semper et al., 2014).

Various studies have found that a large percentage of PD patients test positive for active *H. pylori* [70% *H. pylori*-positive (Dobbs et al., 2012), 53% positive (Lee et al., 2008), 32.4% positive (Tan et al., 2015)]. A study of patients in Denmark reported that patients who had been treated for *H. pylori* infection were at increased risk for developing PD (45% for *H. pylori*-eradication drugs and 23% for proton-pump inhibitors) (Nielsen et al., 2012), suggesting chronic *H. pylori* infection and/or gastritis precede PD symptoms. However, because *H. pylori* is a common pathogen with global distribution, these associations are not particularly surprising.

Because *H. pylori* survival and replication requires local neutralization of the acidic gastrointestinal tract, infection is frequently associated with drug malabsorption, and *H. pylori* infection seems to diminish response to levodopa (L-dopa) therapy. *H. pylori*-infected patients showed a delay in L-dopa onset (the time it take for the drug to “kick in”) versus uninfected patients (Pierantozzi et al., 2001; McGee et al., 2018). Consequently, antibiotic treatment of *H. pylori* led to marked improvement of symptoms in PD patients receiving L-dopa therapy (Hashim et al., 2014). *H. pylori* clearance may improve therapeutic efficacy due to the bacterium's ability to neutralize the pH of the stomach. *H. pylori* secretes urease to break urea down into carbon dioxide and ammonium in order to neutralize stomach acid and create a region of neutral pH around the bacterium. These *H. pylori*-dependent fluctuations in stomach pH are thought to influence the absorption of L-dopa in the duodenum, and there is strong clinical evidence to support this mechanism. For example, higher L-dopa blood serum levels can be measured in *H. pylori*-positive patients treated with antibiotics compared to *H. pylori*-positive patients receiving antioxidant therapy (Pierantozzi et al., 2006). Additionally, PD patients receiving L-dopa following treatment with *H. pylori*-specific antimicrobials have improved hypokinesia (Dobbs et al., 2013). However, to date no study looking at *H. pylori* clearance following antibiotic treatment has assessed the status of patients' gut microflora pre- and post-treatment. Therefore, it is difficult to say definitively whether it is solely the eradication of *H. pylori* that impacts PD symptoms and L-dopa efficacy in antibiotic-treated patients since interplay through the gut-brain-axis is also correlated with PD (Zhu et al., 2017). Indeed, *H. pylori*-dependent alteration of the microbiome and the gut-brain-axis may influence PD outcomes in a number of ways, including altering permeability of the BBB to inflammatory mediators or immune cells (Braniste et al., 2014).

*H. pylori* has also been associated with AD. Kountouras et al. report that AD patients had significantly higher levels of anti-*H. pylori* IgG antibodies in their CSF and serum. While higher levels of anti-*H. pylori* IgG antibodies in the CSF could be an indirect result of changes to the permeability of the BBB, these antibodies also correlated with lower mini-mental state examination scores compared to those of healthy controls (Kountouras et al., 2009). These conclusions remain controversial, however, as recent investigations found no increased risk of AD in *H. pylori*-infected patients (Fani et al., 2018) or only found increased risk in certain population groups, specifically in men and in higher socioeconomic status groups, both of which have higher incidence of complicating co-morbidities like cardiovascular disease (Beydoun et al., 2018).

Interestingly, meta-analyses have demonstrated an opposite trend between *H. pylori* and MS; several studies have found a significantly lower prevalence of *H. pylori* infection in patients with MS (Li et al., 2007; Jaruvongvanich et al., 2016; Yao et al., 2016). The inverse correlation between *H. pylori* and MS is particularly significant in Western countries (Yao et al., 2016). Consistent with these patient studies, pre-infection of C57BL/6 mice with *H. pyl*o*ri* prior to induction of a mouse model of MS (EAE, experimental autoimmune encephalomyelitis) resulted in significantly reduced clinical scores and fewer CD4+, CD8+, Th1, and Th17 cells in the CNS (Cook et al., 2015). Together, these data suggest that some inflammation-inducing pathogens may elicit immune responses that are actually protective for certain CNS diseases, such as in MS where there is an autoimmune component. Although the mechanisms and cell types that drive these divergent outcomes remain unclear, many independent lines of evidence that support a role for *H. pylori* infection in influencing CNS dysfunction.

### Bordetella pertussis

*Bordatella pertussis* is a gram-negative bacterium that causes an infection of the lungs that presents as pertussis, or whooping cough*. B. pertussis* uses a protein called filamentous haemagglutinin to bind to ciliated epithelial cells and colonize the respiratory tract. Its primary mechanism of pathogenesis utilizes a secreted toxin (pertussis toxin, PT) that becomes active after the bacterium is endocytosed into host cells. Once active, PT blocks G-protein signaling, leading to increased cellular concentrations of cAMP and inhibition of cellular signal transduction, impacting the ability of cells to respond to a number of extracellular signals, including hormones, chemokines, and cytokines (Lattin et al., 2007).

While vaccination is incredibly effective in preventing *B. pertussis* infection and spread, incidences of pertussis are high in regions where vaccination coverage is low; a recent modeling study purports there may be as many as 24 million cases of pertussis per year worldwide (Yeung et al., 2017). While vaccination prevents acute clinical pertussis risk, it does not prevent subclinical infection (Ward et al., 2005; Warfel et al., 2014; Zhang et al., 2014), and it is hypothesized that chronic, asymptomatic *B. pertussis* colonization may influence CNS dysfunction. Although there is no evidence for the bacterium itself entering the CNS, researchers have speculated that PT can reach the CNS following absorption from the nasal mucosa. Again, while direct evidence for this popular hypothesis is still lacking, experiments have shown that PT can decrease BBB integrity and promote transmigration of monocytes in a human brain-derived microvascular endothelial cell model of the BBB (Kügler et al., 2007). If these results translate to an *in vivo* infection, this action of PT has the potential to expose the CNS to the toxin, pathogen, or other components of the host immune response.

The immune response to *B. pertussis* infection has a Th1/pro-inflammatory cytokine signature characteristic of innate immune sensing through TLR-4 (i.e., IL-1β, TNF-α, IL-6). When Loscher et al. challenged mice via aerosolized *B. pertussis*, they detected pro-inflammatory cytokines by ELISA in the lungs, circulating plasma, hypothalamus, and hippocampus (Loscher et al., 2000a). The same group detected IL-1β, TNF-α, and IL-6 mRNA in the hypothalamus at various time points following *B. pertussis* infection, suggesting that peripheral infection with this pathogen is sufficient to trigger gene expression in regions of the brain (Loscher et al., 2000b). While the authors argue they can detect cytokine mRNA in infected brains well after bacterial loads in the lungs have cleared (day 42 post-infection), the authors do not formally control for bacteria accessing the brain or other organs.

Chronic asymptomatic infection with *B. pertusiss* has been linked to the development of AD. Experiments in mice have shown that pertussis infection triggers production of Aβ_40_, a proteolytic product of amyloid precursor protein that is an important biomarker for AD (McManus et al., 2014). Specifically, aged AD-model mice [overexpressing APP and harboring mutations in presinilin 1 (*PS1*)] had significantly higher levels of insoluble Aβ_40_ when infected with *B. pertussis* (McManus et al., 2014). *B. pertussis*-infected animals also had more Aβ_40_ plaques, increased T cell infiltration into the brain, elevated macrophage/microglial activation, and increased inflammatory cytokines in brain cortical tissue. Likewise, pertussis toxin itself has been shown to dramatically reduce Aβ_40_ clearance in a mouse microglia cell line (BV-2) (Tahara et al., 2006). Because of the multifactorial nature of *B. pertussis* infection, it is unclear whether PT, the pathogen itself, the immune response, or a combination of these factors, contributes to changes in the CNS.

### Chlamydia pneumoniae

*Chlamydia pneumoniae* is a gram-negative obligate intracellular bacterium and a common cause of upper respiratory infections, including acute pneumonia and bronchitis. *Chlamydial* elementary bodies, which are metabolically inactive but infective, attach to and are phagocytosed by macrophages. *C. pneumoniae* can also infect respiratory epithelial cells in the lungs (Jahn et al., 2000). Once inside a host cell, elementary bodies mature into reticulate bodies, replicate, and differentiate back into elementary bodies, which are released to begin the cycle again. While lysis of host cells is crucial for *C. pneumoniae* spread during acute infection, the bacterium can inhibit apoptosis during chronic infection, allowing for persistence (Rajalingam et al., 2001). As *C. pneumoniae* can infect a variety of cell types, it induces expression of an array of cytokines and chemokines, notably the inflammatory mediators IL-6, IL-1β, IL-8, and TNF-α, and cell adhesion molecules ICAM-1 and VCAM-1 (Kern et al., 2009; Lim et al., 2014).

Despite being primarily categorized as an infection of the lungs, *C. pneumoniae* is also widely associated with cardiovascular disease and atherosclerotic plaque formation. *C. pneumoniae* can damage blood vessels either directly by infecting endothelial cells and vascular smooth muscle cells or indirectly by infecting monocytes/macrophages that damage tissues via inflammation and oxidative bursts (Di Pietro et al., 2017). *C. pneumoniae* is also associated with cerebrovascular disease and cerebral infarction (stroke). A recent meta-analysis suggested that cerebral infarction patients were significantly more likely to be seropositive for *C. pneumoniae* infection compared to healthy controls (Su and Chen, 2014).

Because of its well-established role in pathology outside of the lungs and in plaque formation, it is perhaps unsurprising that connections between *C. pneumoniae* and CNS pathology are beginning to emerge. Several groups have detected *C. pneumoniae* in patient brains by PCR (Arking et al., 1999; Sriram et al., 2005; Gérard et al., 2006). However, the route that *C. pneumoniae* uses to reach the brain remains unclear. Because *C. pneumoniae* is a respiratory infection, both olfactory and lung infection routes are possible. Recent comparative genomics experiments studying strains isolated from or previously identified in the human heart, brain, and lung found that the brain-associated strain (TOR-1) was more similar to the respiratory strain (AR39) than to cardiac-associated strains, suggesting a lung-to-brain migration (Roulis et al., 2015). Such migration could be mediated by infected monocytes capable of crossing the BBB. Ex vivo studies using human brain microvascular endothelial cells (HBMECs) to mimic the BBB, showed that THP-1 monocytic cells infected with *C. pneumoniae* had an enhanced ability to transmigrate through the endothelial monolayer (MacIntyre et al., 2003). Consistent with this, MacIntyre et al. observed an upregulation of adhesion molecules like LFA-1, VLA-4, and MAC-1 in infected monocytes, suggesting that *C. pneumoniae* infection can alter cell-intrinsic qualities of macrophages to enhance their ability to cross the BBB and promote migration into the CNS.

While the mechanism and implications of *C. pneumoniae* brain infiltration remains unclear, associations between *C. pneumoniae* and CNS disorders are quite strong. In one study, *C. pneumoniae* DNA was amplified from 90% of postmortem brains of patients diagnosed with late-onset dementia of the Alzheimer's type, compared to just 5% of healthy control brains (Balin et al., 1998). Another more recent PCR-based study showed that 20/25 AD patients but only 3/27 control patients were PCR-positive for *C. pneumoniae* (Gérard et al., 2006). Using immunohistochemistry, the same study observed that CNS cells harboring *C. pneumoniae* were often found in close proximity to both neurofibrillary tangles and neuritic senile plaques, which are comprised of Aβ_40_ and associated with late-onset AD (Gérard et al., 2006).

*C. pneumoniae* has also been connected to MS. In one landmark study, *C. pneumoniae* DNA was found in the CSF of 36/37 MS patients vs. 5/27 control patients with other neurological disorders. These same MS patients were also more likely to have anti-*C. pneumoniae* IgG (32/37 in MS vs. 0/27 in controls) (Sriram et al., 1999). However, since this initial publication, a number of groups have been unable to culture *C. pneumoniae* from the CSF or brain tissue of MS patients, and additional PCR detection approaches have also been unsuccessful (Gieffers et al., 2000; Ring and Lyons, 2000). Some of these technical difficulties may result from the complicated intracellular lifestyle of *C. pneumoniae*; because it is an obligate intracellular pathogen it cannot freely replicate in a niche like the CSF and is difficult to culture *ex vivo*. Because of these caveats, the presence of *C. pneumoniae* in the CNS of MS patients and the implications of these findings for MS pathogenesis remain divisive.

### Periodontal Bacteria

Periodontitis is a common chronic inflammatory disease caused by a number of oral resident bacteria that leads to inflammation of the gums and damage to teeth themselves. The main bacterial species associated with periodontitis are *P. gingivalis, Tannerella forsythia*, and *Treponema denticola*. All are gram-negative, obligate anaerobes specialized to live in the diverse microbial community of the oral cavity. The immune response associated with periodontitis involves both innate and adaptive immune cells, with inflammatory mediators like IL-1β, TNF-α, IL-6, contributing not only to tissue damage of the gums but also osteoclastogenesis (breakdown of bone tissue) (Cekici et al., 2014).

In recent years, there has been an explosion of studies investigating connections between oral bacteria and neurodegenerative diseases like AD. A retrospective cohort study of patients in Taiwan found that patients with chronic periodontal disease (>10 year exposure) had a 1.707-fold increased risk of developing AD (Chen et al., 2017). GWAS database studies looking at genes in the *P. gingivalis*/host interactome identified significant overlap between host factors and genes involved in cognitive disorders, AD, and dementia. Similar studies also found that the gene expression profiles of macrophages exposed to live *P. gingivalis*, its LPS, or its fimbrae closely matched the expression profiles of hippocampuses from post-mortem AD patients (Carter et al., 2017). Together, these results suggest that periodontal bacteria, and especially *P. gingivalis*, may elicit inflammatory gene expression programs similar to those associated with neurodegeneration.

Evidence for periodontal bacteria in the brain itself remains controversial. To date, most studies of the relationship between periodontal infections and systemic disease have used the *ApoE*^−/−^ mouse model. APOE mediates lipoprotein uptake by the LDL receptor and plays an important role in atherosclerosis and AD. When *ApoE*^−/−^ mice were chronically infected with *P. gingivalis* via oral lavage (repeated inoculations over a course of 12 or 24 weeks), Poole et al. detected bacterial genomic DNA in the brains of 50% of the *ApoE*^−/−^ mice, compared to 0% of the matched controls. The authors speculate that migration of *P. gingivalis* into the brain implicate ApoE in maintaining the integrity of the BBB (Poole et al., 2015). Indeed, *ApoE*^−/−^ mice have been shown to experience increased cortical BBB leakage as they age compared to wild-type controls (Hafezi-Moghadam et al., 2007). Other studies of *P. gingivalis*-infected *ApoE*^−/−^ mice found higher incidence of *P. gingivalis* bacteria in the frontotemporal lobe by fluorescence *in situ* hybridization, which correlated with an increase in age-related granules (astrocyte-associated inclusions observed in the hippocampus of senescence-accelerated mouse models) (Singhrao et al., 2017). In humans, LPS from *P. gingivalis* has been detected in post-mortem AD brain tissue (Poole et al., 2013), and serum IgG levels against *Eubacterium nodatum* and *Actinomyces naeslundii*, two common periodontal bacterial species, were higher in AD patients compared to healthy controls (Noble et al., 2014). A more recent study from Dominy et al. detected *P. gingivalis* and associated virulence proteins called gingipains (RgpB) in the brains and CSF of AD patients. In mice orally infected with *P. gingivalis*, this same group found *P. gingivalis* in 8/8 brains and observed higher levels of the AD marker Aβ_1−42_ in these infected animals (Dominy et al., 2019). Repeated oral application of *P. gingivalis* by Ilievski et al. also led to detection of gingipain in/at brain cells and higher levels of several important inflammatory cytokines (IL-6, TNF-α, IL-1β) (Ilievski et al., 2018).

Additional studies have attempted to link periodontal-associated inflammation more generally to dysregulation of the CNS. In order to investigate the contribution of peripheral inflammatory responses on oxidative stress in the brain, Rokad et al. infected TNF-α transgenic mice with *P. gingivalis*. These mice express a stabilized version of TNF-α mRNA, resulting in overexpression of TNF-α protein in most tissues, including the brain (Rokad et al., 2017). In these mice, *P. gingivalis* infection increased the proportion of oxidized proteins in whole brain lysates, suggesting that *P. gingivalis* may contribute to cellular damage and downstream protein oxidation. In another series of mouse infections, Ding et al. orally infected young (4 week) and middle-aged (12 month) C57BL/6 mice with *P. gingivalis* and tested their learning and memory abilities 6 weeks after infection (Ding et al., 2018). They reported that only middle-aged, *P. gingivalis*-infected mice showed poor performance in a Morris water maze test, while young *P. gingivalis*-infected and all uninfected mice performed equally well. Middle-aged infected mice also exhibited higher levels of pro-inflammatory cytokines (TNF-α, IL-6 and IL-1β) in brain tissue, which is consistent with advanced age being a major risk factor for neurodegenerative diseases. These experiments, when coupled with patient studies, begin to suggest a complicated interplay between periodontal bacteria, neuroinflammation, and CNS dysfunction.

### Borrelia burgdorferi

The ability of spirochetes to infiltrate the brain and cause disease has been well documented in the case of *Treponema palladium*, the causative agent of syphilis and associated neuropsychiatric disorders (Tuddenham and Ghanem, 2018). Less well-understood is how another spirochete, *B. burgdorferi*, the causative agent of Lyme disease, impacts the CNS. *B. burgdorferi* is a motile, microaerophilic, extracellular bacterium transmitted to humans by a tick vector. Containing a thin outer membrane that lacks LPS, *B. burgdorferi* is considered neither gram-negative nor gram-positive. Despite its unusual outer membrane, TLR2 has been implicated in sensing *B. burgdorferi* lipoproteins like OspA, and mice lacking TLR2 have increased bacterial loads (Alexopoulou et al., 2002; Wooten et al., 2002). Activation of innate immune sensing by OspA and other pathogen-associated molecular patterns induces expression of pro-inflammatory products like IL-6, TNF-α, and nitric oxide (NO), as well as immunosuppressors like IL-10 (Chung et al., 2013). Because *B. burgdorferi* is primarily extracellular, B cell responses and antibodies help limit disease and pathogenesis. However, the bacterium has evolved ways to evade neutralizing antibodies, including expression of variable surface antigens and trafficking to the joints and skin where they may be inaccessible to circulating antibodies (Tilly et al., 2008).

Although the precise mechanisms through which *Borrelia* crosses the BBB remain unclear, the detection of *Borrelia* in CSF is well-documented using both PCR and culture methods (Wilske et al., 2007). CNS dysfunction associated with *Borrelia*, often referred to as Lyme neuroborreliosis, occurs during the disseminated stage of the disease and can involve both the central and peripheral nervous system. Curiously, European strains of *Borrelia* are more prone to cause Lyme neuroborreliosis, while North American isolates are more associated with Lyme arthritis, pointing to distinct virulence determinants or co-morbidities that influence disease outcomes (Cardenas-de la Garza et al., 2019). Symptoms of neuroborreliosis (e.g., nerve pain, headache, facial nerve palsy) are generally inconsistent with neurodegenerative disorders, and Lyme neuroborreliosis itself is not associated with neurodegenerative disease. However, there is some, albeit circumstantial, evidence to suggest an association between AD and the presence of *Borrelia* in the brain or CSF.

A case study in 1987 was the first to successfully culture *B. burgdorferi* from brain tissue of a dementia patient with probable early stage AD (MacDonald and Miranda, 1987). More recent studies have detected nucleic acid or antigen evidence of *Borrelia* spirochetes in the CSF, blood, and cerebral cortex of 14 AD patients, while 13 healthy controls were all negative. In three of these cases, *Borrelia* spirochetes were successfully cultured from the brains of post-mortem AD patients (Miklossy et al., 2004). Miklossy et al. have also observed AD-associated neurofibrillary tangles that immunoreact with anti-*B. burgdorferi* antibodies and co-localization of *Borrelia* antigens with Aβ plaques (Miklossy et al., 2004). However, the results of these studies are still being debated, as other groups have been unable to detect *Borrelia* in AD patients' brains using PCR (Marques et al., 2000).

Nonetheless, additional animal and *in vitro* studies have provided support for a link between *Borrelia* and AD. In mouse experiments, labeled lipoproteins from the spirochete *Borrelia turicatae* were detected in the CNS after infection, and pro-inflammatory cytokines were detectable in the brain parenchyma, suggesting that circulating *Borrelia*-derived components may travel to the brain and cause neuroinflammation (Londoño and Cadavid, 2010). In *ex vivo* studies with CNS cells, exposure to *B. burgdorferi* spirochetes can induce Aβ accumulation, tau phosphorylation, and increased AβPP levels, all of which are characteristic of AD (Miklossy et al., 2006). How *B. burgdorferi* induces plaque-like phenomena in these cells is not completely understood. Spirochetes express a number of factors to facilitate their binding to host cells, and many of these factors (e.g., bacterial amyloids, fibronectin- and collagen-binding proteins) can promote protein adhesion and coagulation, perhaps precipitating these AD-like features. Alternatively, it is possible that sensing of spirochetes, or spirochete components, in the brain by resident microglial cells via TLRs and subsequent innate immune activation contributes to neuroinflammation.

## Mycobacterial Infection and PD: A Case Study in Common Molecular Mechanisms Driving Bacterial Pathogenesis and Neurodegenerative Disease

### *M. leprae* and *M. tuberculosis*

*M. tuberculosis* is a non-motile intracellular bacterium that replicates within alveolar macrophages and, during active infection, causes a devastating lung disease. Patients with active *M. tuberculosis* infection present with a pro-inflammatory type I IFN signature, and mice treated with recombinant IFN-β succumb to disease faster than littermate controls (Manca et al., 2001). Likewise, mice lacking the type I IFN receptor (IFNAR) are protected (Stanley et al., 2007; Mayer-Barber et al., 2014), and mutations in IFNAR in human populations also confer resistance to *M. tuberculosis* (Zhang et al., 2018).

Although it is currently the number one infectious disease killer on earth, *M. tuberculosis* only kills a small number of patients it infects (<1%). In the vast majority of cases, *M. tuberculosis* establishes a latent, subclinical infection that can persist for decades. We are only starting to appreciate how the immune system is engaged during latent tuberculosis. Increasingly, research suggests that *M. tuberculosis* is not completely dormant and instead undergoes repeated bursts of replication (Lin and Flynn, 2010). This “percolating” phenotype and its associated cycles of inflammation may impact multiple organ systems, including the CNS, over the lifetime of a patient.

A recent nationwide, population-based cohort study investigating the association between TB and PD purported that patients with TB had a 1.38-fold higher risk of PD compared to the general population (Shen et al., 2016). Conversely, vaccination with neuronal antigen in complete Freund's adjuvant (which contains heat-inactivated *M. tuberculosis*) or vaccination with the tuberculosis vaccine strain Bacilli Calmette-Guerin (BCG) each partially protected against PD-associated neuronal death and prevented microglial activation in the MPTP mouse model of PD (Yong et al., 2011). Together, these data suggest that the immune response to *M. tuberculosis* may trigger or exacerbate CNS dysfunction.

A surprising number of connections between mycobacterial infection and genes associated with neuronal health have recently emerged. Notably, SNPs in several genes, namely *LRRK2, PARK2*, and *PINK1* confer susceptibility to both mycobacterial infection and PD. Genome wide association studies identified these SNPs based on their association with *Mycobacterium leprae*, the causative agent of leprosy. *M. leprae* shares almost all of its genes with *M. tuberculosis*, although it causes distinct pathologies affecting primarily the nerves, skin, and nasal mucosa (Serrano-Coll et al., 2018). The bacterium mainly replicates within macrophages and peripheral nerve cells called Schwann cells. Depending on the host immune response, *M. leprae* causes a spectrum of disease. On one end, patients that develop a strong Th1 response are said to have tuberculoid disease and experience less severe symptoms and clear the bacilli. On the other end of the spectrum, patients with lepromatous disease mount a Th2 response and have high bacterial loads in the skin and nerves accompanied by severe tissue damage (Desvignes and Ernst, 2013). Because Th1/Th2 polarization of the adaptive immune responses is crucial for controlling *M. leprae* pathogenesis, studies frequently identify immune-associated loci (*NOD2, TNFSF15, RIPK2, IL12B*) as leprosy susceptibility determinants (Zhang et al., 2009; Marcinek et al., 2013; Teles et al., 2013). Less clear is why SNPs in PD-associated genes, typically studied in the context of mitochondrial homeostasis in neurons, also confer susceptibility to leprosy. Here, using *M. tuberculosis* and *M. leprae* as case studies, we will explore how molecular connections between PD-associated genes and bacterial pathogenesis may unexpectedly shed light on mechanisms of neuroinflammation and neurodegeneration.

#### Selective Autophagy of Bacteria (Xenophagy) and Mitochondria (Mitophagy) Are Functionally Parallel Pathways

To date, three PD-associated genes have been repeatedly implicated in mycobacterial susceptibility: *LRRK2, PARK2*, and *PINK1*. The proteins encoded by each of these genes have been shown to be important for mitochondrial homeostasis and turnover, which are controlled by a general process known as autophagy. Autophagy is an evolutionarily conserved process in eukaryotes whereby cytosolic components are enveloped and sequestered in double membrane structures that fuse with and are degraded in lysosomes. In response to nutrient limitation, such as amino acid starvation, general autophagy serves a catabolic function by degrading organelles and other cellular components in order to generate substrates for energy metabolism and new protein synthesis. A related process termed selective autophagy renovates the cytosol by specifically tagging damaged organelles and protein aggregates with ubiquitin (colloquially referred to as an “eat me” signal) for their removal. Selective autophagy can mediate turnover of damaged organelles, like mitochondria, in a process known as mitophagy. Selective autophagy also plays an important role in innate immune defense against invading intracellular pathogens, by tagging cytosolically exposed bacteria with ubiquitin and targeting them to autophagosomes (a.k.a. xenophagy).

**Figure 2:**
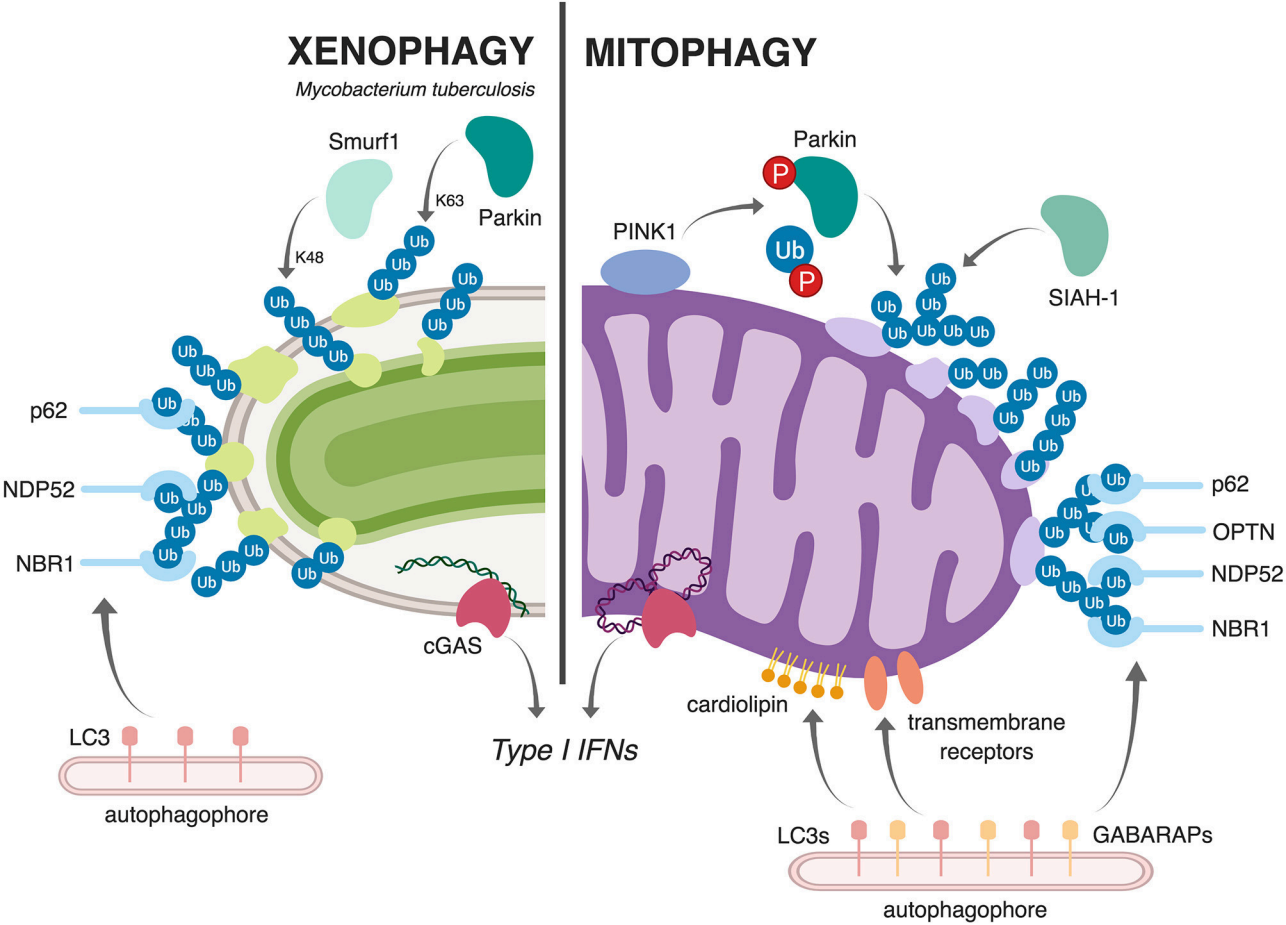
Schematic representation comparing and contrasting mitophagy and xenophagy of Mtb. During xenophagy of Mtb in macrophages **(left)**, Mtb-containing vacuoles are decorated with ubiquitin via the E3 ligases Parkin (Watson et al., 2012) and Smurf1 (Franco et al., 2017). This recruits various selective autophagy adaptors (p62, NDP52, and NBR1) and, subsequently, LC3 and the autophagophore. Upon mitochondrial damage **(right)**, the kinase PINK1 phosphorylates both Parkin and ubiquitin (Kondapalli et al., 2012; Kane et al., 2014; Koyano et al., 2014; Kazlauskaite and Muqit, 2015), allowing Parkin to ubiquitinate mitochondrial proteins. The E3 ligase SIAH1 also contributes to the ubiquitination of mitochondrial proteins (Szargel et al., 2016). As in xenophagy, this ubiquitination recruits selective autophagy adaptors (p62, OPTN, NDP52, and NBR1), LC3, and the autophagophore. Other ubiquitin-independent mechanisms, like cardiolipin-dependent mitophagy and receptor-mediated mitophagy, can also target damaged mitochondria to autophagy; in these pathways, exposed cardiolipin or outer membrane proteins directly recruit LC3 proteins or the closely related GAPARAP proteins. Both Mtb infection and mitochondrial damage activate the cytosolic DNA sensor cGAS **(bottom)**, which elicits a type I IFN transcriptional program (Wassermann et al., 2015; Watson et al., 2015; West et al., 2015; Franco et al., 2017). Figure created with BioRender.

Multiple studies have shown that macrophages use selective autophagy to tag and target a population (~30%) of *M. tuberculosis* bacilli to autophagosomes. These events depend on permeabilization of the *M. tuberculosis* phagosome, which requires the activity of the pathogen's ESX-1 virulence-associated secretion system. As a result of phagosomal permeabilization, the cytosolic DNA sensor cGAS detects bacterial dsDNA and elicits the potent pathologic type I IFN immune response via the cGAS/STING/TBK1/IRF3 signaling pathway (Collins et al., 2015; Wassermann et al., 2015; Watson et al., 2015). Work by Watson et al., demonstrated that *M. tuberculosis*-derived dsDNA also plays an critical role in triggering xenophagy through cGAS/STING, which is required for recruitment of downstream autophagy markers like p62 (Watson et al., 2012, 2015). Other selective autophagy adapters have also been shown to localize to the *M. tuberculosis* phagosome, including NDP52, and NRB1, although it remains unclear whether any of these are absolutely required for autophagosomal targeting or if there is built in redundancy in the repertoire of selective adapters in macrophages. Because evolutionarily, mitochondria were once themselves intracellular bacteria bound by a membrane, it is perhaps not surprising that cells employ similar machinery to sense and clear damaged mitochondria (Manzanillo et al., 2013; West et al., 2015; West and Shadel, 2017). However, as these connections are still new, many questions regarding the parallels between xenophagy and mitophagy persist. We do not fully understand what can serve as “danger signals” to trigger xenophagy and/or mitophagy, and we have a poor understanding of how cells tag these different cargos (bacteria vs. mitochondria) for destruction (e.g., ubiquitinated substrates, sensing molecules, autophagy adaptors, etc). Moreover, the degree to which these pathways share machinery and regulatory components remains unclear. Parallels and differences between mitophagy and xenophagy are illustrated in Figure 2.

#### *PINK1, Parkin*, and *LRRK2* in Mitophagy and PD

Numerous lines of evidence suggest that mitochondrial dysfunction plays a pivotal role in PD. Several mutations linked to PD (*PINK1, PARK2, LRRK2*) affect proteins with critical and well-characterized roles in mitophagy (Table 1). Mitophagy is active in healthy cells to maintain mitochondrial homeostasis but is upregulated in response to various stressors to clear mitochondria that have sustained damage.

**Table 1:** Genes associated with susceptibility with to mycobacterial infection and Parkinson's Disease.

**Gene**	**Relationship to PD**	**Role in mitochondrial homeostasis**	**Reported roles in infection and immunity**	**Implicated in these bacterial infections**
*LRRK2*	Most common genetic cause of PD; responsible for 2% of total PD cases	Important regulation of mitochondrial fission and fusion (possibly though phosphorylation of RAB7) (Beilina et al., 2014) Mutants associated with increased susceptibility to oxidative stress and decreased antioxidant defenses (Angeles et al., 2014) Mutations can activate or inhibit autophagy (Giaime et al., 2017; Mamais et al., 2018) Can physically interact with Parkin (Smith et al., 2005)	Upregulated in response to IFN-y (Gardet et al., 2010) Exogenous expression increases activation of NFκB (Gardet et al., 2010) Required to control *S*. Typhimurium replication in RAW264.7 cells (Gardet et al., 2010) Negative regulator of NFAT (nuclear factor of activated T cells) (Liu et al., 2011) Systemic LPS treatment of LRRK2-mutant mice results in neuronal loss (Schildt et al., 2019) Negative regulator of phagosome maturation in macrophages during *M. tuberculosis* infection (Härtlova et al., 2018) Promotes activation of the NLRC4 inflammasome during *S*. Typhimurium infection (Liu et al., 2017)	*Mycobacterium leprae* (Zhang et al., 2009; Marcinek et al., 2013; Wang et al., 2016) *Mycobacterium tuberculosis* (Härtlova et al., 2018), *Salmonella enterica* serovar Typhimurium (Liu et al., 2017)
*PARK2*(Parkin)	Mutations cause 15% of familial and 4% of sporadic PD cases with onset before 40 years of age Patients with *PARK2* mutations show defects in mitochondrial quality control	Promotes mitophagy by ubiquitinating proteins on the outer membrane of mitochondria following damage/depolarization	Important for controlling *M. tuberculosis* replication in macrophages and in mice, and controlling *M. marinum* and *S*. Typhimurium in *Drosophila*.(Manzanillo et al., 2013) Loss of PARKIN misregulates immune gene expression in *Drosophila* (Greene et al., 2005)	*Mycobacterium leprae* (Mira et al., 2004) *Mycobacterium tuberculosis* (Manzanillo et al., 2013) *Salmonella Typhi* (Manzanillo et al., 2013)
*PINK1*	Mutations increase risk of early onset PD Patients with *PINK1* mutations show defects in mitochondrial quality control	Involved in activation of mitophagy via phosphorylation of Parkin (Kim et al., 2008; Gladkova et al., 2018) Can also mediate Parkin-independent mitophagy (Villa et al., 2018)	Loss of PINK1 impacts mitochondria antigen presentation in mice (Matheoud et al., 2016) PINK1 positively regulates IL-1β signaling through Tollip and IRAK1 (Lee and Chung, 2012)	*Mycobacterium leprae* (Wang et al., 2016)

*For each gene (LRRK2, PARK2, and PINK1), their relationship to PD, role in mitochondrial health, reported roles in infection and immunity, and associated bacterial infections are described*.

Several mechanistic pathways of mitophagy have been described. Most studied is the ubiquitin-dependent mitophagy pathway. When mitochondria are depolarized and cannot import proteins, the kinase PINK1 accumulates on the outer membrane of the mitochondria where it recruits the E3 ligase Parkin (encoded by *PARK2*). PINK1 phosphorylates both Parkin and ubiquitin, activating Parkin to ubiquitinate proteins on the surface of compromised mitochondria. This ubiquitination serves as an “eat me” signal to the cell, and recruits cargo receptors (OPTN, NDP52, p62, and NBR1), which bind to both ubiquitin and the core autophagy protein family LC3. This results in recruitment of the isolation membrane and engulfment of the damaged mitochondrial cargo within an autophagosome. Interestingly, Szargel et al. demonstrated that SIAH, another E3 ubiquitin ligase, is capable of tagging mitochondria with this “eat me” signal, suggesting that E3 ligases in addition to Parkin can participate in ubiquitin-mediated mitophagy (Szargel et al., 2016).

Another mechanism of mitophagy, which is independent of PINK1 and Parkin is called receptor-mediated mitophagy and utilizes transmembrane proteins located on the outer membrane of mitochondria. These transmembrane receptors (Nix/Bnip3, Bnip3, FUNDC1, FKBP8) bind to LC3 proteins as well as the closely related GABARAP proteins to directly recruit autophagosomal membranes. Finally, cardiolipin-mediated mitophagy occurs when cardiolipin, a phospholipid normally found on the inner mitochondrial membrane, is transported to the outer mitochondrial membrane by PLS3 (phospholipid scramblase-3) and hexameric NDPK-D in responses to various types of mitochondrial damage (Chu et al., 2013; Kagan et al., 2016). Once there, cardiolipin serves as another “eat me” signal and is bound directly by LC3 to recruit autophagosomal membranes. Chu et al. found that induction of this pathway is especially robust when neurons are treated with PD-related mitophagy stressors (rotenone, staurosporine, 6-hydroxydopamine), which bolsters the physiological relevance of this pathway in PD progression and further highlights that different cell types and different forms of mitochondrial damage can influence mitophagy. To date, neither receptor-mediated mitophagy nor cardiolipin-mediated mitophagy have been shown to influence bacterial survival or replication in host cells.

The association between PD and gene mutations in mitochondrial quality control pathways has long implied a connection between mitophagy and PD. Gene associations are further supported by patient studies suggesting that environmental toxins that induce mitochondrial stress (such as rotenone, paraquat, MPTP, and other organic chemicals) can increase the risk of sporadic PD. Additionally, age is the largest risk factor for PD, suggesting that accumulation of cellular damage plays a critical role in disease progression and defects in neuronal mitophagy are generally accepted as contributing to PD onset and/or exacerbation. Indeed, neurons have extremely high energy demands in order to maintain ion gradients required for neurotransmission. Since their energy is produced primarily through oxidative respiration, they rely on functional and healthy mitochondria, and dysregulated mitochondrial networks lead to cell death and neuronal defects (Kann and Kovács, 2007). These defects and failure of quality control likely lead to the neuronal loss in the SNc observed in the brains of PD patients.

Mutations in *PINK1* and *PARK2* are the most frequently observed in patients with familial or inherited PD, and *PARK2* mutations are the most common mutation associated with early-onset PD. Loss-of-function mutations in *PINK1* or *PARK2* create a block in mitophagy, resulting in accumulation of damaged and dysfunctional mitochondria. Consistent with these observations, an early study by Zhu et al. showed that mitochondria-containing autophagosomes accumulate in the substantia nigra of PD patients (Kann and Kovács, 2007). Fiesel et al. later showed that there is also a marked increase in phospho-ubiquitin in the substantia nigra of PD patients (Zhu et al., 2003). Accumulation of this PINK1/Parkin-dependent mitophagy marker strongly suggests a pathologic block in this mitophagy pathway as the marked cargo fails to be degraded. However, PINK1 and Parkin also have regulatory roles in the biogenesis of mitochondria via PARIS and PGC1-alpha, so mutations in *PINK1* or *PARK2* could contribute to mitochondrial dysfunction by preventing the replacement of mitochondria (Fiesel et al., 2015).

### *M. tuberculosis* and Parkin

In order for *M. tuberculosis* to be destroyed via xenophagy, it must be targeted with ubiquitin chains. A ubiquitin “cloud” is associated with ~30% of intracellular *M. tuberculosis* bacilli and consists of at least K63- and K48-linked ubiquitin species, suggesting multiple protein substrates are modified with ubiquitin (Manzanillo et al., 2013). Presently, the identity of these protein substrates remains unclear, and there is great interest in illuminating whether ubiquitin chains are conjugated to recruited host signaling proteins or to exposed *M. tuberculosis* surface proteins, or perhaps to both. Similarly, the precise identities of ubiquitinated proteins around damaged mitochondria remain unknown.

Although the nature of ubiquitin targets remains elusive, several groups have identified E3 ubiquitin ligases that play a role in targeting *M. tuberculosis*-phagosomes to xenophagy, including Parkin and Smurf1. In bone marrow derived macrophages, Parkin deficiency coincided with significant decreases in the K63-linked ubiquitin “cloud” around *M. tuberculosis* (Manzanillo et al., 2013). Loss of Parkin also resulted in decreased recruitment of downstream selective autophagy adapters including p62, NBR1, and NDP52. Importantly, *M. tuberculosis* replication was enhanced in both *PARK2*^−/−^ macrophages and in *PARK2* knockdown human monocyte U937 cells, and *PARK2*^−/−^ mice had significantly higher bacterial burdens in their lungs, livers, and spleens compared to wild-type controls. Along these same lines, genome-wide association studies have identified alleles of *PARK2* that increase susceptibility to *M. leprae* (Mira et al., 2004) although the role of xenophagy in controlling *M. leprae* is currently unknown. Susceptibility alleles of Parkin have also been found for *Salmonella Typhi* and paratyphi infection (Ali et al., 2006), the causes of typhoid and paratyphoid fever, respectively, which supports a broad role for Parkin in controlling intracellular pathogens (Table 1).

In a recent paper, Franco et al. showed that the E3 ligase Smurf1 is also required for targeting *M. tuberculosis* to xenophagy. *Smurf1*^−/−^ macrophages were defective in recruiting K48-linked polyubiquitin and NBR1 to *M. tuberculosis*-associated phagosomes, and loss of Smurf1 led to increased bacterial loads *ex vivo* in macrophages and *in vivo* in mice (Franco et al., 2017). Interestingly, Smurf1 has no known role in mitophagy and is only linked to PD by studies examining LPS-induced neuroinflammation, where loss of Smurf1 inhibited neuronal necroptosis in the CNS (Shao et al., 2018). This suggests that certain E3 ubiquitin ligases like Smurf1 may play a privileged role in autophagy following microbial-induced damage and/or inflammation. Together, these data point to critical roles for E3 ligases like Parkin in controlling bacterial replication *ex vivo* and *in vivo* and strongly suggest that human mutations in *PARK2* confer susceptibility to intracellular bacterial pathogens because of defects in ubiquitin-mediated targeting. However, additional roles for Parkin in regulating other immune-relevant cellular pathways can not be discounted.

### *M. tuberculosis* and *LRRK2*

LRRK2 is a large, multi-functional protein, and as such, has many possible roles in regulating mitochondrial dynamics to influence PD pathogenesis. Mutations in this gene occur in both sporadic and familial cases of PD, and it is the most common PD-linked autosomal dominant mutation. Activating mutations in *LRRK2* like G2019S and R144C are common in PD patients, so these mutants have been studied extensively. Such mutations have been shown to increase mitophagy in neuronal cells, which leads to decreased mitochondrial mass, dendrite retraction, and neuronal dysfunction (Cherra et al., 2013). Wang et al. showed that constitutively active LRRK2 mutants exhibit prolonged interactions with Drp1, which promotes mitochondrial fragmentation (Wang et al., 2012), and Hsieh et al. showed that constitutively active LRRK2 mutants are defective in forming a complex with Miro, a protein that anchors mitochondria to microtubules. In the absence of a LRRK2/Miro complex, Miro is stabilized, mitochondria movement proceeds and mitophagy is significantly delayed (Hsieh et al., 2016).

A 2009 New England Journal of Medicine study found that SNPs in *LRRK2* are associated with susceptibility to infection with *M. leprae* (Zhang et al., 2009). Subsequent studies have identified a panel of LRRK2-associated SNPs that associate with leprosy outcome (Wang et al., 2015). A recent report from Harlova et al. showed that loss of *LRRK2* (*Lrrk2*^−/−^) enhanced phagosomal maturation in macrophages, resulting in reduced *M. tuberculosis* replication (Härtlova et al., 2018). *In vivo*, loss of *LRRK2* caused a moderate decrease in *M. tuberculosis* bacterial loads in the lung and spleen of infected mice at early time points. Increased control of *M. tuberculosis* replication correlated with increased expression of several anti-bacterial cytokines (TNF-α, IFN-γ) and lower expression of pro-bacterial IFN-α, although these cytokine differences were seemingly transient. The authors proposed that loss of *LRRK2* enhances *M. tuberculosis* phagosomal maturation, but how these cell-intrinsic differences translate to enhanced innate immune cytokine expression remains unclear. Surprisingly, the authors found no LRRK2-dependent changes to xenophagy of *M. tuberculosis*, despite a number of reports linking LRRK2 to mitochondrial turnover in other cell types (Manzoni, 2012). Curiously, Harlova et al. also reported that macrophages harboring the PD-associated G2019S gain-of-function LRRK2 mutation supported higher levels of *M. tuberculosis* growth. The differing involvement of LRRK2 in xenophagy vs. mitophagy could be due to cell-specific LRRK2 functions or unknown differences between targeting of bacterial pathogens vs. damaged mitochondria (for example, ubiquitin-mediated vs. receptor- or cardiolipin-mediated). Together, these studies imply an important but complex role for LRRK2 in controlling intracellular *M. tuberculosis* replication as well as in regulating systemic immune responses and inflammation during infection (Table 1).

### *M. tuberculosis* and *PINK1*

Based on GWAS studies showing association between SNPs in *LRRK2* and mycobacterial infection, the same group looked for SNPs in other mitochondrial/PD-associated genes including PINK1. In their study of Han Chinese from South China, Wang et al. reported *PINK1* SNP rs4704 was associated with increased susceptibility to leprosy (Wang et al., 2016; Table 1).

The mechanism by which PINK1 impacts leprosy susceptibility remains a mystery and to date, no one has reported on the requirement for PINK1 in controlling *M. tuberculosis* infection. It is possible that PINK1 plays a direct role in initiating the tagging and targeting of damaged *M. tuberculosis* and/or *M. leprae*-phagosomes to xenophagy, mirroring its role in mitophagy. It is also conceivable that PINK1, along with Parkin and LRRK2, plays important indirect roles in regulating innate immune responses to mycobacterial infection. For example, accumulation of damaged mitochondria can increase levels of ROS and induce low-level activation of type I IFN expression downstream of cytosolic DNA sensing of mtDNA leaked from damaged mitochondria (West et al., 2015). Because type I IFN levels in large part dictate the outcome of *M. tuberculosis* infection, it is likely that maintaining healthy mitochondria in immune cells is important for controlling bacterial pathogenesis in the periphery. Therefore, discovering precisely how these PD-associated genes contribute to both cell-intrinsic and inflammatory responses will expand our understanding of how immune responses to bacterial infection might lead to neurodegeneration.

### Neuroinflammation, Bacterial Infection, and Neurodegeneration: Outstanding Questions

Discerning correlation from causation is the major hurdle in defining a role for infection in triggering neurodegeneration. It is impossible to control or prevent human exposure to pathogens, and many infections are ubiquitous in the population. Therefore, concluding that a specific infection precipitates a particular neurodegenerative disease is almost impossible. The odds are that most PD, AD, and MS patients will have been exposed to a number of the bacterial pathogens reviewed here, as well as viral pathogens associated with neurodegenerative diseases, over the course of a lifetime. A recent epidemiological study tested this “multiple microbe” hypothesis and indeed reported that PD risk was increased in individuals who were seropositive for five or six of the pathogens queried (CMV, EBV, HSV-1, *B. burgdorferi, C. pneumoniae*, and *H. pylori*), compared to healthy controls. The emerging idea that mutations associated with CNS dysfunction may themselves impact susceptibility to infection further exacerbates this already complex “chicken or egg” conundrum.

Looking to the future, our best bets for studying the role of bacterial pathogens in neuroinflammation and neurodegenerative diseases are likely animal models of infection and controlled cellular studies. A number of pathogens described here, including *B. burgdorferi, Mycobacterium tuberculosis*, and *H. pylori*, have well-established mouse models of infection that mimic human disease in many ways. By combining bacterial and mouse genetics, we might begin to tease apart whether a.) bacterial pathogens themselves directly mediate CNS damage, b.) immune responses to bacterial infection mediate CNS damage, and/or c.) CNS mutations or CNS dysfunction influence susceptibility to bacterial infection. For example, infections could be performed in knockout mice that lack key immune receptors/cytokines to distinguish bacteria- vs. immune-related CNS phenotypes. Such immune receptor mutations could be targeted to specific cell types (e.g., macrophages, microglia, neurons) to distinguish how cells in the periphery vs. cells in the brain contribute to neuroinflammation. Likewise, bacterial mutants lacking key virulence factors could be used to modulate distinct outcomes in terms of tissue damage, dissemination, and immune outcomes. Infections could also be performed in wild-type mice alongside mice predisposed to certain CNS disorders or mice with mitochondrial defects to simulate a “multiple-hit” scenario. Ideally, groups that have expertise in both bacterial pathogenesis and neuroinflammation will undertake these experiments so that conditions that best mimic human disease can be maintained (e.g., natural routes of inoculation and low-dose inoculum).

Cellular studies allow for further control over experimental variables compared to animal models but carry many caveats. Previous approaches have depended on overexpression of key proteins, non-physiological methods of mitochondrial damage, and monocultures of cell types with varying *in vivo* relevance. Many of these studies have reported conflicting observations depending on cell type and experimental conditions. Indeed, it is difficult to mimic human disease conditions *in vitro* with cellular conditions, since physiologic stresses are subtler and occur sometimes repeatedly over many decades of life. Moving forward, it will be important to investigate how mutations in PD-associated genes impact mitochondrial health, gene expression, and the metabolic state of not only neurons but also cell types in the periphery, such as macrophages, T cells, B cells, etc.

Along these lines, there are many outstanding questions regarding how factors related to mitophagy and xenophagy impact bacterial pathogenesis and neurodegeneration. For some time, researchers have been puzzled by the lack of overt PD phenotypes in mice harboring mutations in PD-associated genes. Recently, Pickrell et al. found that Parkin knockout coupled with mitochondrial DNA stress precipitated PD symptoms (Pickrell et al., 2015), and Sliter et al. showed that PINK1 knockout combined with exhaustive exercise or mitochondrial DNA stress induced PD-like phenotypes. Interestingly, loss of STING rescued the PD symptoms, indicating type I IFN-related inflammation plays a key role in this process (Sliter et al., 2018). Together, these studies provide experimental support for the multiple-hit hypothesis, and imply that patients with mutations in PD-linked genes need an additional stressor, such as inflammation, to trigger PD progression (Newman and Shadel, 2018). If this type of PD-associated mitochondrial damage is precipitated in an immune cell like a macrophage, one would expect dysregulation of innate immune gene expression, as mtDNA is a known agonist of cytosolic DNA sensors and the type I IFN response (West et al., 2015; West and Shadel, 2017). Consistent with this idea, mutations in *LRRK2* have been shown repeatedly to impact innate immune gene expression in macrophages *ex vivo* and control caspase-1 activation and IL-1β secretion via the NLCR4 inflammasome (Liu et al., 2017). Such dysregulation of the innate immune response could be a consequence of prolonged pathogen sensing and/or mitochondrial damage and mtDNA leakage, both of which would result from defects in mitophagy/xenophagy-related pathways.

Future work should build upon these exciting new perspectives so that we might fully uncover the molecular mechanisms that drive CNS changes during infection. Such discoveries will identify infection-dependent biomarkers that can be correlated with disease outcomes and open the door for novel immune-targeted therapeutic interventions designed to halt or slow neurodegenerative disease progression.

## Author Contributions

KP conceived the structure and content of the manuscript. KP, SB, and CW contributed content. KP, SB, and RW edited final manuscript.

### Conflict of Interest Statement

The authors declare that the research was conducted in the absence of any commercial or financial relationships that could be construed as a potential conflict of interest.
